# Obstructive Sleep Apnea: A Case Report

**DOI:** 10.7759/cureus.38644

**Published:** 2023-05-06

**Authors:** Mohithan Subramaniam, Karthik Rajaram Mohan, Saramma Mathew Fenn, Ravikumar Pethagounder Thangavelu

**Affiliations:** 1 Oral Medicine and Radiology, Vinayaka Mission's Sankarachariyar Dental College, Vinayaka Mission's Research Foundation, Salem, IND

**Keywords:** drug induced sleep endoscopy, polysomnography, nocturnal snoring, uvula, obstructive sleep apnea (osa)

## Abstract

Obstructive sleep apnea (OSA) is characterized by repeated episodes of upper airway blockage and collapse during sleep accompanied by awakenings with or without oxygen desaturations. During obstructive sleep apnea events, the oropharynx in the back of the throat compresses, causing arousal, oxygen desaturation, or both, leading to fragmented sleep. The hyperplastic uvula is a common clinical finding in patients with obstructive sleep apnea. The various diagnostic and treatment modalities of obstructive sleep apnea are discussed in this article.

## Introduction

Repeated episodes of upper airway obstruction and collapse during sleep, followed by awakenings with or without oxygen desaturations, are the hallmarks of obstructive sleep apnea (OSA). During obstructive sleep apnea (OSA) events, the oropharynx in the back of the throat compresses, causing arousal, oxygen desaturation, or both, leading to fragmented sleep [1]. The risk factors for obstructive sleep apnea include increased obesity, body mass index (BMI), increased hip-waist ratio, chronic smokers, obese people with a BMI over 35, people with congestive heart failure, atrial fibrillation, treatment-resistant hypertension, type 2 diabetes, stroke, nocturnal dysrhythmias, pulmonary hypertension, high-risk driving populations (like commercial truck drivers), and people undergoing bariatric surgery are all examples of high-risk patients [1,2].

## Case presentation

**Figure 1:**
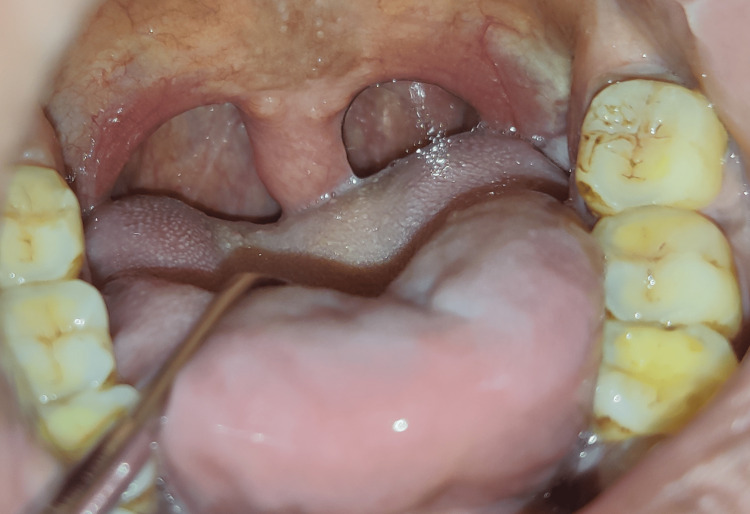
Intraoral clinical photograph revealed a hyperplastic uvula

A 46-year-old male reported to our department with a chief complaint of the feeling of irritation in the throat for the past six months. On eliciting a history, the patient revealed that he has episodes of disturbed sleep and wakes suddenly from sleep due to choking and difficulty breathing while sleeping at night. A general examination revealed his vitals are stable and afebrile. Extraoral examination revealed no cervical lymphadenopathy. On intraoral examination, his uvula appeared long and increased in size (Figure1). The Mallampati score was an independent predictor of obstructive sleep apnea, which describes the visual evaluation of the space available for intubation procedure during general anesthesia based on the distance between the base of the tongue and the roof of the mouth. It is a deceptive method of determining how challenging intubation is. The Mallampatti score, in our case, was assessed as Class II (in which soft palate, fauces, and a major part of the uvula are visualized) [3].

The tip of the uvula was visualized only when the patient was asked to pronounce the word "ah" during clinical examination with a mouth mirror [VIDEO 1].

**Video 1:** Intraoral clinical examination of hyperplastic uvula with an aid of mouth mirror

**Figure 2:**
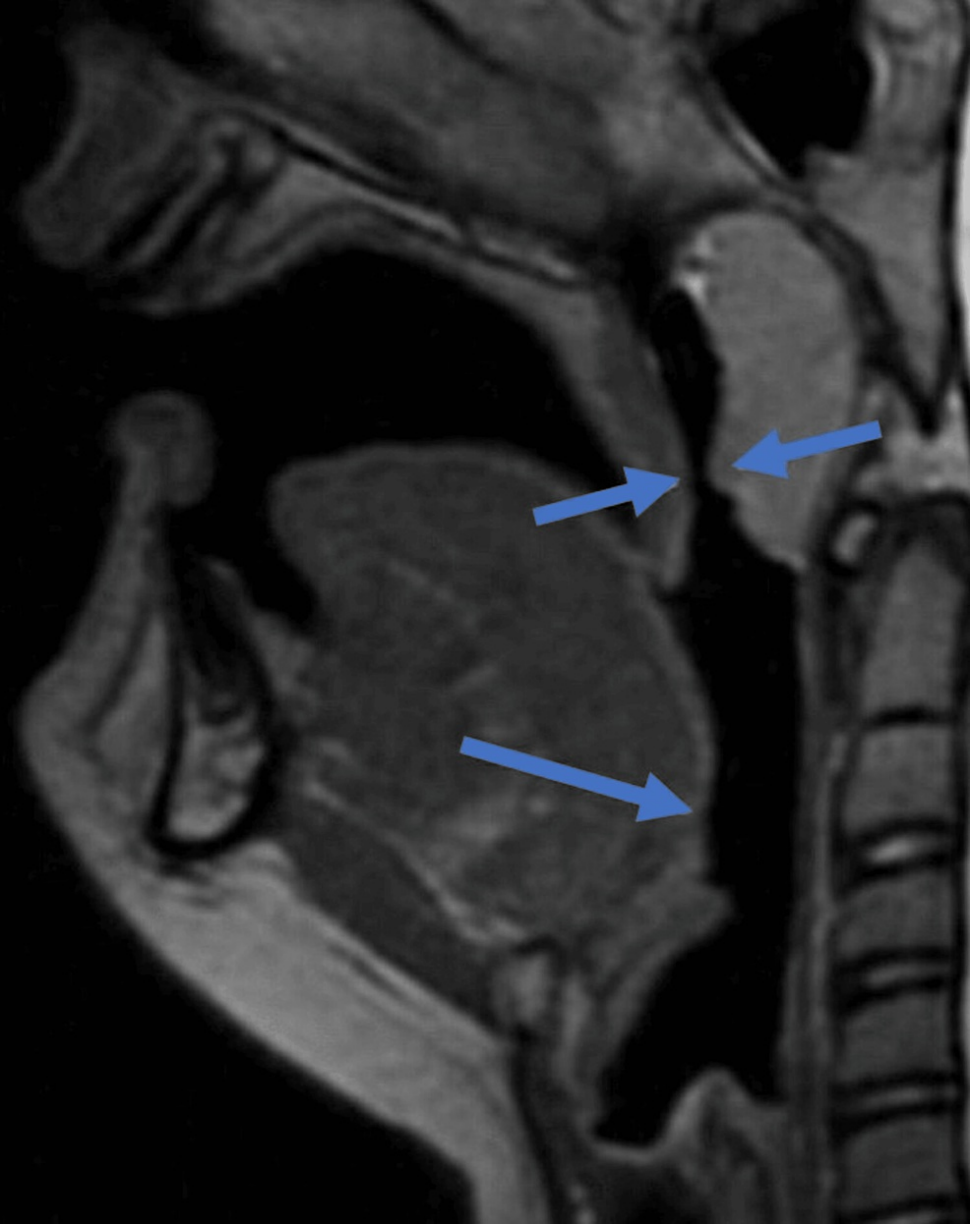
MRI- Sagittal section revealed long uvula that constricts the airway spaces

Laboratory investigations such as glycosylated hemoglobin revealed HbA1C = 5.2% ( less than 5.5 indicates good glycaemic control over diabetes or absence of diabetes), tri-iodothyronine (T3) = 1.42 nmol/L (normal range = 1.2-3.0 nmoL/L, thyroxine (T4) = 84 nmol/L (normal range = 70-140 nmol/L) and thyroid-stimulating hormone (TSH) = 3.9 mU/L (normal ranges = 0.5-5.7 mU/L) were also assayed to rule out hypothyroidism, which showed normal biological ranges. The sagittal section of magnetic resonance imaging (MRI) revealed a long uvula resting on the tongue's posterior part and a narrowing of pharyngeal air spaces (Figure 2).

Correlating the history of the feeling of irritation in the throat for the past six months and the history of disturbances during sleep due to choking and the presence of hyperplastic uvula, a provisional diagnosis of obstructive sleep apnea due to hyperplastic uvula was made. The differential diagnosis includes epidermoid cysts affecting the uvula and human papillomavirus-induced papillary hyperplasia of the uvula. The epidermoid cysts affecting the uvula are developmental and gradually increase in size during the growth period of the individual. Papillomas affecting the uvula usually have a tiny finger or cauliflower-like projections on the surface of growth with seropositivity for the human papillomavirus. Narcolepsy is a chronic sleep disorder characterized by extreme daytime sleepiness and unexpected sleep bouts. Regardless of the situation, people with narcolepsy frequently struggle to stay awake for extended periods. The patient denied surgical treatment.

## Discussion

Definition

Obstructive sleep apnea (OSA) is characterized by repeated episodes of upper airway obstruction and collapse during sleep, followed by microarousals with or without oxygen desaturations, which are the hallmarks of obstructive-sleep apnea (OSA) [1]. Snoring and/or increased respiratory effort as a result of increased upper airway resistance and pharyngeal collapsibility are symptoms of obstructed sleep-disordered breathing (SDB), which is not a distinct illness. Instead, it is a syndrome of abnormal upper airway function when you sleep. A sleep disorder called obstructive sleep apnea (OSA) also referred to as obstructive sleep apnea-hypopnea, causes the airflow to halt or drastically decrease even when breathing is being actively worked. Obstructive sleep apnea is characterized by a considerably diminished (hypopnea) or absent (apnea) airflow at the nose and/or mouth during sleep as a result of the upper airway's increased collapsibility. Oxygen hemoglobin desaturation is commonly followed by a transient micro-arousal, which are brief recurrent minor awakening or arousal lasting for 1.5 to 3 seconds [3].

Synonyms

Apnea/hypopnea syndrome, upper airway resistance sleep apnea syndrome, obstructive sleep apnea-hypopnea, and Pickwickian syndrome also known as the obesity-hypoventilation syndrome [4].

Epidemiology

According to apnoea-hypopnea index (AHI) standards, the prevalence of obstructive sleep apnea (OSA) is 7.5% in urban India and around 3.73% in rural India. The term "apnea" refers to the recurrent episodes of complete cessation of airflow through the nostrils, and "hypopnea" refers to the diminished airflow through the nostrils that occur in patients with obstructive sleep apnea. In obstructive sleep apnea, the "apnea-hypopnea index" is the number of times apnea and hypopnea happen simultaneously regularly. Men were affected 50% and females 25% in the middle-age group. The prevalence of obstructive sleep apnea (OSA) increases to 36.34 million people when extrapolating these statistics to the 974.3 million rural residents of India [2,3]. The prevalence rate of obstructive sleep apnea was reported in other countries by meta-analytic studies [4] [Table 1].

**Table 1:** Various research studies on obstructive sleep apnea [4]

Author	Year	Place of study	Sample size	OSA Diagnostic tool	Average age (In Years)	Prevalence
Andayeshgar B et al.	2022	Iran	10754	Meta-analytical study	58.6+/-4.1	56%
Vishwanathan et al.	2017	Chennai	203	Device	54	23.6 %
Amin et al.	2017	Europe	129	Device	59.3	75.2%
Donavan et al.	2017	Israel	818	Questionnaire	63.5	90.3%
Sokwalla et al.	2017	Kenya, Africa	223	Questionnaire	56.8	44.4 %
Zhang et al.	2016	China	880	Device	60.5	60%
Westlake et al.	2016	Prague	294	Device	64.7	72%
Nasseri et al.	2015	Iran	173	Questionnaire	55.4	54%
Sadeghnilat et al.	2015	Iran	173	Questionnaire	61.6	74%
Zhang et al.	2015	China	337	Device	54.5	66.7%
Obaseki et al.	2014	Nigeria	117	Questionnaire	63	27%
Burgess KR	2013	Queensland and New South Wales	1109	Device	53	71%
Lecome et al.	2012	France	3894	Questionnaire	66	85%
Lam et al.	2010	Hong Kong	824	Device	57.4	53.9%

Classification of obstructive sleep apnea

During obstructive sleep apnea (OSA) events, the oropharynx in the back of the throat compresses, causing arousal, oxygen desaturation, or both, leading to fragmented sleep [3]. The American Academy of Sleep Medicine classifies the severity of obstructive sleep apnea as described below [2] (Table 2).

**Table 2:** Types of obstructive sleep apnea based on Apnea-Hypopnea Index by American Academy of Sleep Medicine

Types of Obstructive Sleep Apnea (OSA)	Characteristics	Apnea-Hypopnea Index (AHI)
Mild	Involuntary sleepiness during activities that require little attention, such as watching Television (TV) or reading	5 to 15 events per hour
Moderate	Involuntary sleepiness during activities that require some attention, such as meetings or presentations	15 to 30 events per hour
Severe	Involuntary sleepiness during activities that require more active attention, such as talking or driving	>30 events per hour

Risk factors

The various structural and non-structural risk factors for obstructive sleep apnea are described in Table 3 [3].

**Table 3:** Risk factors for Obstructive sleep apnea

Risk factors for obstructive sleep apnea	
Structural risk factors	Micrognathia, retrognathic maxilla or mandible resulting from maxillary or mandibular hypoplasia (immature or below normal size due to arrested state or lack of complete development), adenoids (enlarged palatine tonsil) in children and young adults, high arched palate.
Non-structural risk factors	Chronic smoking, obesity, central fat distribution in the neck, advancing age, post-menopausal women, chronic alcohol use or opioids.

Symptoms

Obstructive sleep apnea symptoms include loud snoring that occurs frequently, observable apneas, restless sleep, nocturia, and mouth breathing. The observed apneas during sleep are the distinguishing feature of obstructive sleep apnea [3]. Some daytime signs could be: Sleep that does not restore energy (such as "rising just as exhausted as when they went to bed"), headache, dry or sore throat in the morning, excessive drowsiness during the day (EDS), which typically starts during quiet activities, daytime drowsiness or tiredness, memory and intellectual disability; cognitive deficiencies, and sexual problems, including lowered libido and impotence. Unwanted snoring is annoying and can accurately predict 71% of sleep-disordered breathing (SDB). The specificity of disruptive snoring and documented apneas for sleep-disordered breathing (SDB) is 94% [3].

Diagnostic modalities for obstructive sleep apnea

The various diagnostic modalities for obstructive sleep apnea are discussed in Table 4.

**Table 4:** Types of sleep studies and diagnostic modalities for obstructive sleep apnea

Types of Sleep studies	Diagnostic modalities for obstructive sleep apnea
Type 1 (Level 1)	Polysomnography (PSG): The gold standard for diagnosis of obstructive sleep apnea. Multimodal analysis that measures cardiorespiratory and neurologic parameters during sleep.
Type 4 (Level 4)	Hypnogram: A hypnogram is a polysomnography that displays the various sleep stages over time, including wakefulness, stages 1, 2, and 3 of sleep, and rapid eye movement (REM) sleep. It also includes waveforms for other parameters like body position, respiratory events (such as apnea and hypopnea), microarousals, continuous positive airway pressure therapy, and oxygen saturation. Home sleep apnea Tests (HSAT): The home sleep apnea tests (HSAT) monitor 4 to 7 parameters, including oximetry, effort (inductive plethysmography), and airflow (thermal and nasal pressure). Sleep is not tracked with an electroencephalogram. The best candidates for this therapy are adult patients who are at high risk for moderate to severe obstructive sleep apnea (OSA) based on the STOP-BANG (snoring, tired, observed, pressure, body mass Index, age, neck-size, gender) questionnaire or who have day time sleepiness and two of the three symptoms of snoring detected apnea or hypertension.
Drug-induced sleep endoscopy (DISE)	Drug-induced sleep endoscopy (DISE): Endoscopic visualization of upper airway structures helps to identify the structure that causes obstruction.
Apnea-hypopnea index (AHI)	Apnea-hypopnea index (AHI): The near complete cessation of airflow for 10 seconds is called apnea. Hypopneas refers to a partial decrease in airflow for 10 seconds or respiratory-effort-related arousals or subtle changes in airflow due to increased upper-airway resistance that results in arousals. Apnea-hypopnea index (AHI) measures the number of apneas or hypopneas that occur during sleep divided by the sleep time in hours.

Screening questionnaires for obstructive sleep apnea

Table 5 shows the various questionnaires used for assessing the severity of obstructive sleep apnea [3].

**Table 5:** Questionnaires for screening obstructive sleep apnea

Screening questionnaires for obstructive sleep apnea
Epworth sleepiness scale (ESS): Epworth sleepiness scale (ESS) helps to assess obstructive sleep apnea subjectively. To assess a patient's subjective level of sleepiness, the Epworth Sleepiness Scale is frequently employed in sleep medicine. The test asks you to score your propensity to nod off in eight situations on a scale from 0 (no likelihood of nodding off) to 3 (high chance of nodding off). Then, a scale from 0 to 24 calculates your final score. The ranking determines whether you feel overly sleepy and may need medical intervention.
Berlin Questionnaire (BQ): The Berlin questionnaire consists of 3 categories related to the risk of having sleep apnea. Three items on the Berlin questionnaire deal with the possibility of experiencing sleep apnea. Patients can be categorized as High Risk or Low Risk based on their responses to the individual items and their aggregate scores in the symptom categories.
STOP-Bang Questionnaire (SBQ): The STOP-Bang Questionnaire comprises the following yes/no questions on following: Snoring? Do you Snore Loudly (loud enough to be heard through closed doors or your bed-partner elbows you for snoring at night)? Tired (T)? Do you often feel tired, fatigued, or sleepy during the daytime (such as falling asleep during driving or talking to someone)? Observed? Has anyone observed you stop breathing or choking/gasping during your sleep? Pressure? Do you have or are being treated for high blood pressure? Body Mass Index more than 35 kg/m^2^? Age older than 50? Neck size large? (Measured around Adam’s apple) Is your shirt collar 16 inches/40cm or larger? Gender = Male? For the general population, obstructive sleep apnea (OSA): Low Risk: Yes to 0-2 questions; Intermediate Risk: Yes to 3-4 questions; High Risk: Yes to 5-8 questions or Yes to two or more of 4 STOP questions + male gender or Yes to two or more of 4 STOP questions + Body Mass Index (BMI) > 35kg/m^2^ or Yes to two or more of 4 STOP questions + neck circumference 16 inches/40cm. The highest sensitivity in a sleep clinic scenario for identifying obstructive Sleep apnea in high-risk patients.
Modified STOP Questionnaire Snoring? Do you snore loudly (loud enough to be heard through closed doors or your bed-partner elbows you for snoring at night)? Tired (T)? Is your Epworth Sleepiness Scale Score (a measure of tiredness) >or = 10 observed? Has anyone observed you stop breathing or choking/gasping during your sleep? Pressure? Do you have or are being treated for high blood pressure?

Medical management of obstructive sleep apnea

Dronabinol: A nonselective agonist of the cannabinoid type I and type II receptor, it has been shown to lower central apneas and the apnea-hypopnea (AHI) index. The recommended dosage for the treatment of obstructive sleep apnea is 2.5-10 mg/day [2].

Modafinil: Modafinil has been approved by the US Food and Drug Administration (FDA) for use in patients who still feel sleepy during the day despite using continuous positive airway pressure (CPAP) to the fullest extent possible. Patients who used modafinil at 200-400 mg/d daily doses reported the greatest improvements. Unknown is the modafinil's wakefulness-inducing mode of action. Its wake-promoting effects are comparable to those of sympathomimetic drugs [2].

Armodafinil: The R-enantiomer of modafinil, armodafinil, 250 mg per day, has just received US Food and Drug Administration (FDA) approval for its use in patients with obstructive sleep apnea [2].

Surgical and non-surgical treatment modalities for obstructive sleep apnea

The various surgical and non-surgical modalities for obstructive sleep apnea [4-13] (Table 5).

**Table 6:** Non-surgical and surgical treatment modalities for obstructive sleep apnea

Treatment modalities for obstructive sleep apnea		
	Medications	Tab. Modafinil 200 mg once daily in the morning to prevent early morning sleepiness in Obstructive sleep apnea. Tab. Armodafinil 250 mg once daily (FDA-approved drug) for obstructive sleep apnea Tab. Dronabinol 2.5 to 10 mg per day to lower episodes of apnea and hypopnea in obstructive sleep apnea. But the main disadvantage was drug addiction.
Non-surgical modalities	Mini implant-assisted Rapid Maxillary Expansion (MARME)	For adults with obstructive sleep apnea. Patients with skeletal transverse maxillary deficiency enable more significant separation of mid palatal suture area and an increase in the volume of the nasal cavity.
Surgical modalities	Uvulopalatopharyngoplasty (UPP)	For obstructive sleep apnea caused by oropharyngeal obstruction. It involves surgical removal of the uvula, tonsils, and posterior velum.
	Genioglossus advancement with hyoid myotomy or suspension	For obstructive sleep apnea caused by the base of tongue obstruction.
	Tracheostomy	Only considered as a last resort procedure. Advantage: It bypasses upper airway obstruction.
	Maxillomandibular advancement surgery	Enlarged dimensions of the upper airway in anteroposterior and lateral dimensions combine standard Le Fort I osteotomy with a mandibular sagittal split osteotomy for the advancement of the maxilla and mandible. The base of the tongue and soft palate are drawn forward, which increases the airway space and reduces upper airway resistance.
	Modified Maxillomandibular advancement surgery (MMMA)	Segmental maxillomandibular rotational advancement technique (extrusion of the anterior segment, elongation of the posterior maxilla, and counter-clockwise rotation of the maxillomandibular complex) performed in an Asian population with convex craniofacial profile and dentoskeletal Class II malocclusion.
	one-stage multi-level surgery with modified Z-Palatoplasty (ZPP) with one-layer closure, CO2 laser partial tongue-base glossectomy, and bilateral septomeatoplasty	Reduces more apnea than hypopnea and also converts some apnea to hypopnea.
	Neurostimulation	Hypoglossal nerve stimulation. Beneficial in patients with BMI less than 50.

Thiol-disulphide is an essential redox parameter and is assessed as a screening tool in obstructive sleep apnea to monitor affected patients, and such parameters are increased when compared to healthy unaffected individuals [14]. Obstructive sleep apnea was successfully controlled using DIORS OAm (an oral appliance with mandibular advancement), a novel technology that used the Camper plane as a reference for disocclusion [15]. Transoral epiglottopexy, also known as glossoepiglottopexy, is the least invasive surgical procedure available for treating patients with obstructive sleep apnea caused by epiglottis closure. It also has the lowest risk of complications and postoperative morbidity [16,17]. Bleeding (2.6%), candidiasis (0.3%), dryness (7.2%), dysgeusia (0.3%), dysosmia (0.2%), globus sensation (8.2%), surgical site infection (1.3%), velopharyngeal (VP) insufficiency (3.9%), and VeloPharyngeal stenosis (1.6%) are the percentages and associated complications of laser-assisted uvulopalatoplasty (LAUP) [18]. Local anesthesia may be used during laser-assisted uvulopalatoplasty (LAUP) and radiofrequency-assisted uvulopalatoplasty (RAUP) procedures. The CO2 laser is most frequently used in LAUP, while some authors also advocated the use of NdYAG and KTP lasers in the treatment of obstructive sleep apnea (Shiffman HS, Khorsandi J, Cauwels NM 2021). To shield the posterior pharyngeal wall from the laser beam, a backstop should be introduced. On either side of the uvula, triangular incisions are made to remove extra mucosa. The procedure is repeated several times to get the desired result. RAUP is carried out using a radiofrequency generator. Similar to this, triangular incisions are made into the soft palate on either side of the uvula. When necessary, the redundant posterior arch, soft palate, and uvula mucosa are removed; this process is repeated until the desired result is obtained [19]. NightLase® LAUP is a minimally invasive outpatient laser-assisted uvulopalatoplasty surgery that is a safe and effective treatment option for obstructive sleep apnea [20].

Novelty 

The case describes a unique cause of hyperplastic uvula that caused obstructive sleep apnea. The case report enlightens us about the importance of early diagnosis and screening of patients with obstructive sleep apnea, its diagnostic, and non-surgical and surgical treatment modalities.

Limitations

Post-treatment photographs and follow-up are not done, as the patient refused treatment.

## Conclusions

Obstructive sleep apnea is still a commonly neglected, ubiquitous medical health problem. Obstructive sleep apnea though common, typically goes undiagnosed. Loud snoring, nocturnal awakenings, and daytime tiredness are symptoms of obstructive sleep apnea. An increase in the awareness of the consequences of obstructive sleep apnea among the public is essential. The barriers to diagnosis and treatment have significantly decreased in recent years thanks to strategic treatment planning with a multidisciplinary approach by the clinicians, which made it easier for primary care doctors to manage obstructive sleep apnea (OSA) early. More research studies are needed in this field of sleep medicine to manage obstructive sleep apnea in the near future.
